# Hematological Indices of Sickle Cell Anaemia Patients with Pulmonary Tuberculosis in Northern Nigeria.

**DOI:** 10.4084/MJHID.2010.014

**Published:** 2010-06-07

**Authors:** Sagir G. Ahmed, Audu A. Bukar, Bashir Jolayemi

**Affiliations:** 1 Department of Haematology, Aminu Kano Teaching Hospital, PMB 3452, Kano, Kano State, Nigeria; 2 Department of Haematology, University of Maiduguri Teaching Hospital, PMB 1414, Maiduguri, Borno State, Nigeria; 3 Department of Haematology, Federal Medical Center, PMB 1022, Birnin Kudu, Jigawa State, Nigeria

## Abstract

Nigeria has the fourth highest prevalence of TB and the highest prevalence of Sickle cell anaemia (SCA) in the world. SCA patients have impaired immunity and are vulnerable to TB. Hence, we studied the haematological indices of SCA patients with TB in Nigeria. A total of 23 SCA patients with TB were studied in parallel with equal number of age and sex matched SCA patients without TB. SCA patients with TB had significantly lower haematocrit, higher level of circulating sickle cells (CSCs) and similar level of reticulocyte count in comparison to patients without TB. SCA patients with TB had significantly higher mean WBC count associated with higher frequency of neutrophilia in comparison to those without TB. Monocytosis and eosinopenia were exclusively found in SCA patients with TB at frequencies of 52% and 65% respectively. Lymphocyte and basophil counts were normal in all patients with and without TB. Mean platelet counts were high in both patient groups but the frequency of thrombocytosis was significantly higher in patients with TB. SCA patients with TB had significantly higher mean ESR than their counterparts without the infection. The findings of this study revealed that TB in SCA patients was associated with rising level of CSCs, falling level of haematocrit, sub-optimal reticulocytosis, neutrophilia, monocytosis, thrombocytosis, eosinopenia and rising level of ESR. Hence, SCA patients presenting with these haematological indices should be investigated for TB.

## Introduction:

Sickle cell anaemia (SCA), which is the prototype of the sickling diseases, is the homozygous state for the sickle cell gene.^1^ The clinical presentation of SCA is due to vaso-occlusive episodes resulting from polymerization of deoxygenated haemoglobin-S leading to the formation of sickled red cells.^2^ The clinical course of SCA is typically characterized by variable periods of steady state that is periodically interrupted by vaso-occlusive crises.^3^ Although red cell sickling is more prominent during crisis, continuous sickling does occur at a lower rate in the steady state.^4^ Hence, a certain proportion of sickled cells are always present in the circulation of SCA patients even in steady state. Circulating sickled red cells are usually detectable on blood films where they constitute important diagnostic hallmarks.^5^

With a population of over 140m, Nigeria has the world’s fourth largest tuberculosis (TB) burden, lagging behind only India, China and Indonesia.^6^ The annual incidence of new cases of TB was estimated to be over 300 cases per 100,000 population in Nigeria.^6^ Like in many other countries in Africa, TB has attained high level of prevalence as a result of the vicious interactions between poverty, ignorance and high prevalence of HIV infections resulting in many cases of co-infections.^7^ The high prevalence of TB in Nigeria creates a large infection reservoir that increases the risk of contracting the infection even among immune competent individuals, the risk being higher for those with HIV and non-HIV associated immunodeficiency states. Patients with sickle cell anaemia have significantly impaired immunity and hence would be at increased risk of contracting TB.^8^ Nigeria is the most populous black nation that carries a heavy disease burden due to SCA, which affects about 2% of the general population.^9^ Hence, from global perspective, Nigeria has more SCA patients than any other country in the world.^9^ It is therefore imperative to document salient haematological features that maybe associated with TB in patients with SCA in order to facilitate early detection of cases among this vulnerable group of patients. In this paper we studied the haematological indices of adult patients with SCA and pulmonary TB in northern Nigeria.

## Materials And Methods:

This study is a five-year prospective study conducted during year 2004 at the Federal Medical Centre, Birnin Kudu, northwest Nigeria, during the years 2005 to 2007at University of Maiduguri Teaching Hospital, Maiduguri, northeast Nigeria and during the year 2008 at Aminu Kano Teaching Hospital, Kano, northwest Nigeria. In each hospital, patients were recruited from the adult haematology clinic. All subjects studied were diagnosed as SCA based on positive sickling tests and haemoglobin electrophoresis at a pH of 8.6 on cellulose acetate paper.^10^ Consecutive patients with SCA who presented at the clinics in steady state with variable combinations of features of pulmonary TB (cough, haemoptysis, fever, weight loss, dyspnoea, abnormal breath sounds on auscultation, radiographic consolidations) with positivity for acid fast bacilli by sputum microscopy using Ziehl-Neelsen stain were recruited into the study. Only anti-TB treatment naïve cases were included in the study. Equal number of age and sex matched patients with SCA in steady state without features of any infections were recruited as controls. Exclusion criteria for both cases and controls include positive tests for HIV and hepatitis C antibodies and hepatitis B surface antigen. Patients that received blood transfusion within the past 3 months were also excluded.

The haematocrit, white blood cell (WBC) and platelet counts were performed using automatic blood analyzers (Celltac Alpha MEK 6400, Beckman Coulter AcT diff and Cell-Dyn 3700CS). The reticulocyte counts were performed manually using brilliant cresyl blue supravital stain.^11^ The levels circulating sickled cells (CSCs) were estimated from Leishman stained blood films that were examined under microscope (with x100 oil immersion objective) and CSCs were identified and counted as percentages of the total red cells enumerated in the field.^12^ Nucleated red cells were also enumerated on the blood films and automated WBC count errors were manually corrected. Erythrocyte sedimentation rates (ESR) were determine using the Westergren method.^13^ All procedures were conducted with due consent of the subjects and institutional ethics committee approval.

The mean and standard deviation of the haematological parameters were determined for SCA patients with and without TB. Statistical analyses were performed using computer soft ware SPSS version 11.0 and comparisons between the two patient groups were based on Student’s t-test and a p-value of less than 0.05 was taken as significant.

## Results:

A total of 23 cases of SCA with pulmonary TB and equal number of SCA patients without pulmonary TB were studied. The demographic and haematological data accrued from this study is as shown on **Table 1**. Patients with TB had mean haematocrit (0.021L/L) that was significantly lower than the corresponding values of 0.27L/L found among patients without TB (p<0.05). Patients with TB had mean levels of CSCs of 9.5% that was significantly higher than the corresponding value of 4.5% found among patients without infection (p<0.5). However, the mean reticulocyte counts for patients with TB (11%) and without TB (10%) were not significantly different (P>0.05). Patients with TB had mean levels of WBC (13.5 x10^9^/L), neutrophils (9.4 x10^9^/L), monocytes (0.8 x10^9^/L) and platelets (562 x10^9^/L) counts that were significantly higher than the corresponding values of 11.2 x10^9^/L, 7.5 x109/L, 0.4 x10^9^/L and 463 x10^9^/L found among patients without TB (p<0.05). Neutrophilia (neutrophil count >7.5 x10^9^/L) was seen in 19 (83%) and 14 (61%) of SCA patients with TB and without TB respectively (p<0.05). Monocytosis (monocyte count > 0.8 x10^9^/L) was seen in 12 (52%) of patients with TB; monocytosis was not seen in patients without TB. The mean values of lymphocyte and basophil counts seen in patients with TB (3.2 x10^9^/L, 0.04 x10^9^/L) and those without TB (3 x10^9^/L, 0.05 x10^9^/L) were not significantly different (p>0.05). Lymphocyte and basophil counts were normal in all patients with and without TB. Patients with TB had mean eosinophil count (0.08 x10^9^/L) that was significantly lower than the corresponding values of 0.2 x10^9^/L found among patients without TB (p<0.05). Eosinopenia (eosinophil count < 0.04 x10^9^/L) was seen in 15 (65%) of patients with TB; eosinopenia was not seen in patients without TB. Thrombocytosis (platelet count> 450 x10^9^/L) was seen 23 (100%) of patients with TB and 20 (87%) of patients without TB (p<0.05). Patients with TB had mean levels of ESR (57mm/h) that was significantly higher than the corresponding value of 18mm/h found among patients without infection (p<0.05). Elevated ESR (>12mm/h) was found in all patients with TB and without TB.

## Discussion:

The pattern of haematocrit in this study revealed that our subjects suffered from mild to moderately severe anaemia. The presence of CSCs and reticulocytosis in both patient groups was indicative of on-going background red cell sickling and haemolysis, a finding that was consistent with previous reports that sickling occurs in SCA patients even in steady state.^4^ However, the study revealed a significantly lower haematocrit levels among SCA patients with TB as compared to those without the infection. This finding was consistent with the relatively higher level of CSCs among patients with TB. The higher levels of CSCs found among patients with TB was interpreted to be due to impaired pulmonary function leading to greater hypoxia and increased red cell sickling, which led to higher rate of haemolysis and the observed lower levels of haematocrit. Nonetheless, the mean reticulocyte count of SCA patients with TB was similar to that found among the uninfected patients despite the fact that patients with TB had significantly lower haematocrit. This pattern of inadequate reticulocyte response to anaemia among the infected patients was indicative of sub-optimal erythropoietin production consistent with the pathologic process associated anaemia of chronic infections as previously reported in patients with TB.^14^,^15^ Therefore, pulmonary TB in SCA patients was associated with increased severity of anaemia due to combined effect of increased rate of steady state haemolysis and anaemia of chronic infection.

The finding of elevated WBC counts in SCA patients with and without TB was consistent with earlier studies, which showed that leucocytosis was a common feature of SCA even in steady state in the absence of infections and was thought to be due to redistribution of granulocytes from marginal to circulating pool.^16^,^17^ However, our data revealed significantly higher mean WBC count among SCA patients with TB. That was attributed to higher frequency of neutrophilia among the SCA patients with TB in comparison to those without TB. Moreover, a high proportion of SCA patients with TB had monocytosis that was not seen in the non-infected patients. These findings were consistent with previous studies in which neutrophilia and monocytosis were seen in patients with TB.^18^,^19^ The relatively lower eosinophil counts seen among the infected patients was interpreted to be a reflection of the inflammatory response to TB, which was consistent with the considerably higher ESR values seen among the infected patients. Even though eosinopenia was typically associated with acute inflammations and acute phase reactions due to acute bacterial infections, our findings would suggest that chronic inflammations due to TB could also cause eosinopenia.^20^,^21^ However, mildly elevated ESRs were also found among our uninfected patients. This was thought to be due to anaemia as low haematocrit is generally associated with elevation of ESR.^13^ Furthermore, the absence of eosinopenia in the uninfected patients was consistent with the absence of infectious or inflammatory causes of ESR elevation in that group of patients.

Thrombocytosis was found in all patients with TB and the majority of those without TB. Thrombocytosis in SCA patients was attributed to the background haemolytic anaemia and autosplenectomy.^17^,^22^ However, our data revealed that SCA patients with TB had higher frequency of thrombocytosis and significantly higher mean platelet count than their counterparts without TB. This finding was interpreted to be a reflection of additional effect of reactive thrombocytosis due to the inflammatory changes associated with TB.^23^,^24^

The clinical implications of the haematological changes found in SCA patients with TB are two-fold. Firstly, worsening anaemia with an attenuated reticulocyte response would increase the transfusion requirements of affected patients thereby increasing the risk of acquiring transfusion transmissible infections. Secondly, the intensification of leucocytosis, thrombocytosis and levels of CSCs within a background inflammatory acute-phase response would lead to higher blood viscosity thereby amplifying the inherent risks of vaso-occlusion and thrombo-embolism associated with SCA.^25^,^26^,^27^ There is therefore the need for early identification of infected cases by diligent evaluation of haematological parameters so as to ensure that SCA patients with suggestive indices are promptly subjected to definitive microbiological investigations and early treatment.

## Conclusion:

Pulmonary TB in SCA patients was associated with rising level of CSCs, falling level of steady state haematocrit, sub-optimal reticulocytosis, neutrophilia, monocytosis, thrombocytosis, eosinopenia and rising level of ESR. Hence, SCA patients infected with TB should be monitored hematologically since they have a high probability for thrombosis and vaso-occlusive crisis.

## Figures and Tables

**Table 1.** Demographic and Haematological Parameters of Sickle Cell Anaemia Patients with and without Pulmonary Tuberculosis.

**PARAMETERS**	**PATIENTS WITH T.B.**	**PATIENTS WITHOUT T.B.**	**STATISTICAL SIGNIFICANCE**
Mean Age	27	25	
Sex Ratio (Male/Female)	13/10	11/12	
Haematocrit (L/L) Mean ± SD	0.21 ± 0.02	0.27 ± 0.03	P<0.05
Level of CSCs (%) Mean ± SD	9.5 ± 1.2	4.5 ± 0.6	P<0.05
Reticulocyte Count (%) Mean ± SD	11 ± 1.5	10± 1.2	P>0.05
WBC Count (x10^9^/L) Mean ± SD	13.5 ± 3.5	11.2 ± 3.3	P<0.05
Neutrophil Count (x10^9^/L) Mean ± SD	9.4 ± 3	7.5± 2	P<0.05
Lymphocyte Count (x10^9^/L) Mean ± SD	3.2 ± 0.25	3 ± 0.3	P>0.05
Monocyte Count (x10^9^/L) Mean ± SD	0.8 ± 0.12	0.4 ± 0.1	P<0.05
Eosinophil Count (x10^9^/L) Mean ± SD	0.08 ± 0.03	0.2 ± 0.06	P<0.05
Basophil Count (x10^9^/L) Mean ± SD	0.04 ± 0.01	0.05 ± 0.02	P>0.05
Mean Platelet Count (x10^9^/L) Mean± SD	562 ± 48	463 ± 45	P<0.05
ESR (mm/h) Mean ± SD	57 ± 4	18± 2	P<0.05
